# Predicting New-Onset Atrial Fibrillation in Hypertrophic Cardiomyopathy: A Review

**DOI:** 10.3390/jcm14062018

**Published:** 2025-03-16

**Authors:** Marco Maria Dicorato, Paolo Basile, Maria Ludovica Naccarati, Maria Cristina Carella, Ilaria Dentamaro, Alessio Falagario, Sebastiano Cicco, Cinzia Forleo, Andrea Igoren Guaricci, Marco Matteo Ciccone, Vincenzo Ezio Santobuono

**Affiliations:** 1 Interdisciplinary Department of Medicine, University of Bari “Aldo Moro”, Polyclinic University Hospital, 70124 Bari, Italy; m.dicorato20@studenti.uniba.it (M.M.D.); paolo.basile@uniba.it (P.B.); marialudovica97@libero.it (M.L.N.); m.carella31@phd.uniba.it (M.C.C.); ilaria.dentamaro@gmail.com (I.D.); a.falagario2@studenti.uniba.it (A.F.); cinzia.forleo@uniba.it (C.F.); andreaigoren.guaricci@uniba.it (A.I.G.); marcomatteo.ciccone@uniba.it (M.M.C.); 2 Internal Medicine Unit “Guido Baccelli”—Arterial Hypertension Unit “Anna Maria Pirrelli”, Department of Precision and Regenerative Medicine and Jonic Area (DiMePReJ), University of Bari “Aldo Moro”, Polyclinic University Hospital, 70124 Bari, Italy; sebastiano.cicco@uniba.it

**Keywords:** hypertrophic cardiomyopathy (HCM), atrial fibrillation (AF), electrocardiogram (ECG), left atrial remodeling, echocardiography, cardiovascular magnetic resonance (CMR), machine learning, genetics, heart failure, atrial cardiomyopathy

## Abstract

Hypertrophic cardiomyopathy (HCM) is a condition characterized by left ventricular hypertrophy, with physiopathological remodeling that predisposes patients to atrial fibrillation (AF). The electrocardiogram is a basic diagnostic tool for evaluating heart electrical activity. Key electrocardiographic features that correlate with AF onset are P-wave duration, P-wave dispersion, and electromechanical delay in left atrium (LA). Clinical markers, including age, body mass index, New York Heart Association functional class, and heart failure symptoms, are also strong predictors of AF in HCM. Risk scores have been created using multiple variables to better predict AF development. Increasing knowledge of genetic subsets in HCM and cardiovascular pathology in general has provided novel insight in this context. Structural and mechanical LA remodeling, including fibrosis, altered LA function, and changes in atrial size, further contribute to AF risk prediction. Cardiovascular magnetic resonance (CMR) and echocardiographic measures provide accurate information about atrial structure and function. Machine learning models are increasingly being utilized to refine risk prediction, incorporating a wide range of variables. This review highlights the multifaceted approach required to understand and predict AF development in HCM. Such an approach is imperative to enhance prognostic accuracy and improve the quality of life of these patients. Further research is necessary to refine patient outcomes and develop customized management strategies for HCM-associated AF.

## 1. Introduction

Hypertrophic cardiomyopathy (HCM) is a cardiac condition characterized by a left ventricular (LV) wall thickness ≥15 mm in any myocardial segment that cannot be attributed solely to loading conditions, such as arterial hypertension, coronary heart disease, valvular disease, or congenital heart disease [1,2]. In first-degree relatives, the diagnosis is defined by a LV wall thickness of 13 mm or greater. HCM is the most frequent form of cardiomyopathy, with an estimated prevalence of 0.2% in the adult population [2], and a global impact on both sexes [1]. In the patient population with HCM, approximately 40% to 60% exhibit an identifiable pathogenic or likely pathogenic variant [3]. The clinical manifestations of the disease exhibit a wide spectrum, ranging from asymptomatic forms to heart failure, systolic dysfunction, atrial and ventricular arrhythmias, and sudden cardiac death [4,5]. Atrial fibrillation (AF) has been identified as the most prevalent sustained arrhythmia in patients with HCM [6]. Symptomatic paroxysmal episodes have been documented in approximately 20% of these patients. Individuals affected by HCM exhibit a six-fold increased risk compared to the general population [1,7,8]. AF is characterized by the loss of efficient atrial contraction and atrioventricular synchrony. Consequently, its acute onset is poorly tolerated in patients with HCM, necessitating prompt intervention to restore sinus rhythm [9]. AF is associated with a high risk of cardioembolic events and heart failure, significantly influencing morbidity and mortality [10]. Thus, in this population, it is crucial for clinicians to identify potential risk factors for AF onset, in order to perform appropriate screening and early diagnosis, consequently improving prognosis [1]. The objective of this review is to elucidate the electrocardiographic, clinical, genetic, and imaging predictors of AF onset in individuals affected by HCM.

## 2. Electrocardiographic Markers

**Figure 1:**
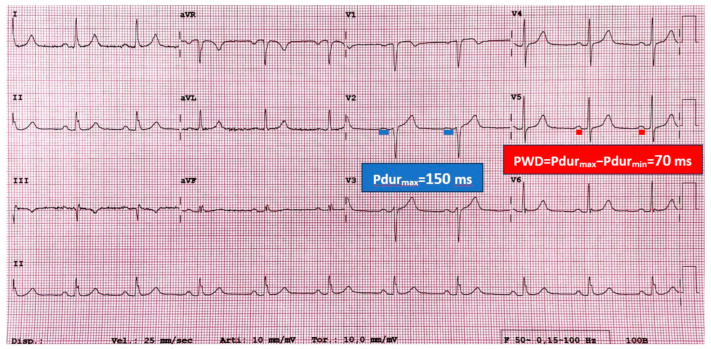
The image shows the sinus rhythm electrocardiogram (ECG) of a 60-year-old patient affected by hypertrophic cardiomyopathy (HCM), who developed atrial fibrillation during the follow-up. P-wave electrocardiographic features are measured. Maximum P-wave duration (Pdurmax) is 150 milliseconds (ms), recorded in lead V2. P-wave dispersion (PWD) is given by the difference between Pdurmax and minimum Pdur (Pdurmin), which is calculated in lead V5 in this case. PWD is calculated as follows: PWD = Pdurmax − Pdurmin = PdurV2 − PdurV5 = 150 ms − 80 ms = 70 ms. Higher values of Pdurmax and PWD have been demonstrated to be associated with atrial fibrillation onset in HCM patients. This ECG explains how ECG data can contribute to the prediction of atrial fibrillation in HCM.

The electrocardiogram (ECG) is a fundamental technique used in the evaluation of patients affected by cardiomyopathies and, specifically, HCM [11,12]. Due to its affordability and ease of implementation, it is employed as the primary diagnostic instrument in the setting of cardiologic disease, offering valuable diagnostic and prognostic insights [3]. In the context of sinus rhythm, the electrical impulse originates in the sinoatrial node and propagates into the atrial myocardium through the conduction myocardium. It has been well established that the prolongation of intratrial and interatrial conduction times, along with the irregular propagation of sinus impulses, are electrophysiological features of an atrium prone to AF. These features can be assessed by ECG analysis. Two electrocardiographic parameters provide data regarding the impulse distribution properties: P-wave duration (Pdur) and P-wave dispersion (PWD) [13]. Pdur is calculated for each lead as the time (expressed in milliseconds) elapsed between the onset and the end of the P-wave, assessing interatrial conduction. PWD is defined as the absolute difference between the maximum and minimum durations of the P-wave [14], and its elevation suggests a heterogeneous atrial conduction. The prognostic value of PWD in predicting AF onset was investigated in a cohort of HCM patients by Cecchi et al. Their study demonstrated that a Pdur ≥ 140 milliseconds (ms) in sinus rhythm electrocardiograms was effective in identifying HCM patients who were likely to develop AF [15]. Because Pdur assessment is impractical, it is not widely used in routine clinical evaluations. Consequently, the measurement of the maximum P-wave duration (Pdurmax) and of PWD is regarded as a superior approach. In a retrospective study encompassing 80 patients with HCM, Ozdemir et al. appraised these two parameters as prognostic indicators of AF occurrence. The authors observed that a Pdurmax value > 134.5 ms and a PWD value > 52.5 ms were capable of differentiating patients with AF from those in sinus rhythm, with adequate sensitivity and specificity [16]. Girasis et al. conducted an evaluation of P-wave duration in a cohort of 30 patients diagnosed with HCM and at least one paroxysmal AF episode. This population exhibited significantly higher left atrial (LA) antero-posterior diameter, reduced atrium peak strain rate in the reservoir phase, and prolonged P-wave duration (with a cut-off value of 98.8 ms) when compared with HCM controls [17]. In a prospective study including 126 patients, Mandeş et al. demonstrated that PWD, differently from LA size, was an independent predictor of AF in patients with HCM and an antero-posterior LA diameter < 45 mm [14]. In this study the cut-off value for PWD was 47.5 ms, in agreement with the value found in previous research by Tuluce et al. [18]. This evidence suggests that early alterations in electrical activation and propagation of the action potential in the LA play a pivotal role in the pathogenesis of AF [19,20] (Figure 1). An increase in P-wave duration is associated with a prolongation of interatrial conduction time, even before an actual enlargement of the LA occurs, thus representing an early marker of AF onset [21]. ECG alterations are strongly correlated with LA mechanical remodeling as well. Indeed, Tjahjadi et al. assessed the electromechanical delay in the LA in a cohort of 208 HCM patients, considering the interval between the onset of the P-wave on the ECG and the peak of the a’ wave (PA interval) in the LA lateral wall, measured by tissue Doppler. They demonstrated that an increase in PA interval duration was independently associated with the onset of AF [22]. The identification of electrocardiographic features that can predict AF development with significant accuracy in patients affected by HCM is a valuable and cost-effective tool, representing a mandatory first-line diagnostic assessment in the clinical path of these patients.

## 3. Clinical Markers

The relationship between specific clinical parameters and AF occurrence is well acknowledged in the general population. A multitude of studies have sought to identify independent clinical predictors of AF, even in patients affected by HCM. Initial research has indicated a correlation between age and AF, both in the general population and in patients with HCM, likely attributable to prolonged exposure to elevated left ventricular filling pressure [8,23,24]. In this regard, Olivotto et al. found in a cohort of 480 HCM patients, followed for a median period of 9 years, an occurrence of AF in 22% of them. The arrythmia was independently predicted by advancing age, congestive heart failure symptoms, and LA enlargement at diagnosis. Although its occurrence was predominant in patients >60 years, it was even documented at earlier ages. AF occurrence was found to be independently associated with adverse outcomes, including HCM-related mortality, stroke, and severe functional impairment [8]. Maron et al. identified age ≥ 40 years as a significant independent risk factor for AF occurrence in a large HCM population [25]. In a similar vein, Klopotowski et al. found that individuals aged above 44.5 years exhibited an increased risk of developing AF [26]. In the context of the Hypertrophic Cardiomyopathy Registry, which included 2755 patients, Kramer et al. found that age was a significant predictor of major AF endpoints, defined as electrical cardioversion, catheter ablation, hospitalization > 24 h, or clinical decision to accept permanent AF. The results showed additional significant predictors of AF, including obesity, moderate-to-severe mitral regurgitation, and a history of arrhythmias. Each of the aforementioned parameters was age-dependent. The study also evidenced that body mass index (BMI) was a robust predictor of AF in younger patients, while, in middle-aged and elderly groups, advanced age was the main cause of LA structural and contractile impairment [27]. Maron et al. conducted a prospective study on 900 HCM patients, evidencing through multivariate analysis that stroke and other peripheral vascular events were independently associated with congestive symptoms and advanced age, as well as with AF [24]. Another parameter that has been widely evaluated as a predictor of AF in patients with HCM is the New York Heart Association (NYHA) functional class, which is considered a clinical indicator of heart failure (HF) severity [28,29]. Cochet et al. studied a group of 209 HCM patients and identified NYHA class as an independent predictor of AF development, along with LA volume and the presence of fibrosis at LV-RV insertion points [28]. The NYHA classification has been incorporated into different clinical scoring systems designed to predict the onset of AF in patients with HCM. The “HCM-AF score” has been recently developed and validated. It has been demonstrated to predict the two- to five-year risk of AF onset in individuals with HCM. The score incorporates the following variables: left atrial dimension, age at clinical evaluation, age at initial HCM diagnosis, and HF symptoms [30]. The occurrence of AF is associated with an increased risk of cerebral thrombotic events as well [31]. Consequently, Guttman et al. created and validated a risk score for cerebrovascular accidents (CVA) in individuals affected by HCM. This score, termed “HCM-CVA Risk”, is a complex, weighted score that includes age, the presence of AF, the age of AF onset, previous thromboembolism, NYHA class, LA size, maximal LV wall thickness, and vascular disease [32]. Building upon these findings, Fauchier et al. developed the “French HCM score”, which incorporates additional clinical features such as diabetes, AF, chronic kidney disease, history of cancer, previous stroke, HF with congestion, and age. In HCM patients without AF, both the HCM-CVA Risk and the French HCM score exhibited efficacy in predicting the risk of ischemic stroke [33]. More recently, artificial intelligence-based algorithms have been employed in this context. Battacharya et al. presented the first machine learning model to predict AF onset in HCM, called the “HCM-AF-Risk Model”, which included a total of 18 variables, both clinical and non-clinical, such as age, NYHA functional class, and dyspnea on exertion [34]. Currently, none of these scores have been included in European guidelines; thus, further studies in this context are needed. However, the findings discussed above underscore the importance of establishing the clinical profile of HCM patients to predict their risk of AF occurrence, which is increased at baseline compared to the general population.

## 4. Genetic Implications

The onset of AF in HCM is typically attributed to increased afterload, which leads to structural remodeling of the LA [35]. A genetic susceptibility to AF has been postulated due to its elevated prevalence in certain families with HCM. Indeed, this phenomenon cannot always be ascribed just to hemodynamic factors [36,37]. Genetic conditions have been shown to directly augment vulnerability to primary atrial cardiomyopathy and, consequently, to AF [38,39]. Moreover, the efficacy of AF ablation is significantly diminished in this patient population compared to those without HCM, indicating the presence of additional arrhythmic substrates [40]. Gruver et al. conducted a study on 24 patients with HCM who were affected by the missense mutation Arg663His in the β-cardiac myosin heavy chain (β-MHC) gene. The study revealed that 47% of the patients experienced AF during a seven-year follow-up period. This percentage was significantly higher compared to that of ungenotyped familial HCM populations [36]. A study by Olivotto et al. revealed that individuals with sarcomere myofilament mutations exhibited a more unfavorable prognosis compared to those with myofilament-negative mutations [41]. Furthermore, patients with myofilament-positive HCM exhibited an elevated risk of cardiovascular death, nonfatal stroke, NYHA class deterioration, and LV dysfunction, independent of age. While there was no significant difference in the incidence of AF between the two groups, the chronic form of the arrhythmia was more prevalent in patients with myofilament-positive HCM. In a subsequent cohort study, Bongini and colleagues investigated the correlation between specific genetic mutations associated with HCM and the risk of developing AF. The study population was divided into three subgroups based on specific gene mutations, with similar LA size: MYBPC3, MYH7, and “other genotypes”, including TNNT2, TNNI3, TPM1, and MYL2. Contrary to prior research, the authors found that genotype was not correlated to AF onset or severity. Instead, the results suggested a more prominent role played by hemodynamic determinants of atrial dilatation [42]. It is noteworthy that AF manifested earlier in the “other genotypes” group, suggesting distinct molecular pathways for AF development in this population. Additional studies have explored the potential involvement of non-sarcomeric genes in the pathogenesis of AF. Among the other molecules, aldosterone has been identified as a key player in atrial structural and electrical remodeling, increasing collagen synthesis and cardiomyocyte apoptosis. The renin–angiotensin–aldosterone system (RAAS) has been demonstrated to contribute to the development of ventricular hypertrophy through the effects of circulating angiotensin, as well as through its local activation within the myocardium [43]. Aldosterone synthesis is predominantly governed by RAAS [44] via a mitochondrial cytochrome P450 enzyme, aldosterone synthase (CYP11B2). Conversely, activated fibroblasts are responsible for type I collagen deposition in all tissues, and excessive synthesis of this type of collagen results in fibrosis. Indeed, the transcription of the type I collagen gene (COL1A1) is 3- to 10-fold increased in activated fibroblasts [45,46]. In a study by Orenes-Piñero et al., specific polymorphisms in the aforementioned non-sarcomeric genes, CYP11B2 (344 T > C) and COL1A1 (2046 G > T), were examined in a population of 159 HCM patients and 136 controls. This specific CYP11B2 polymorphism independently predicted the onset of AF [47], and was associated with higher aldosterone serum levels. On the other hand, the presence of the polymorphism 2046 G > T in the COL1A1 gene showed a non-significant trend towards a lower risk of AF onset, suggesting a potential protective role. Belenkov et al. examined various polymorphisms in different RAAS-linked genes in HCM patients, demonstrating that the 1166A/G polymorphism of AGTR1 was a predictor of AF development [48]. This evidence emphasizes the significant role of non-sarcomeric genes in the phenotypic heterogeneity of HCM (Table 1). Recent studies [49,50,51,52,53] also suggest that AF developing in younger patients could be the initial manifestation of an atrial cardiomyopathy caused by rare variants in genes associated with cardiomyopathies or arrhythmia syndromes. However, the current guidelines do not recommend routine genetic testing in this category of patients [54]. Genetic testing has become pivotal in the clinical management of HCM and cardiomyopathies, as assessed by European Guidelines [2]. Indeed, it allows exact identification of pathogenic mutations, enabling early diagnosis, even in asymptomatic individuals. Moreover, genetic testing gives information about prognosis, since specific gene mutations lead to worse outcomes, and informs treatment decisions [2,55]. Genetic testing also plays a crucial role in identifying at-risk family members, facilitating reproductive management, personalized monitoring strategies, and pre-symptomatic interventions [2]. For all the aforementioned reasons, it has become a key tool in the clinical management of these patients. Genetics has also demonstrated the potential to elucidate the mechanisms underlying the onset of AF in HCM patients. However, further investigation, encompassing larger patient cohorts and genetic subsets, is necessary to more thoroughly explore these correlations.

## 5. Structural and Mechanical Left Atrial Remodeling Detected by Imaging Techniques

**Figure 2:**
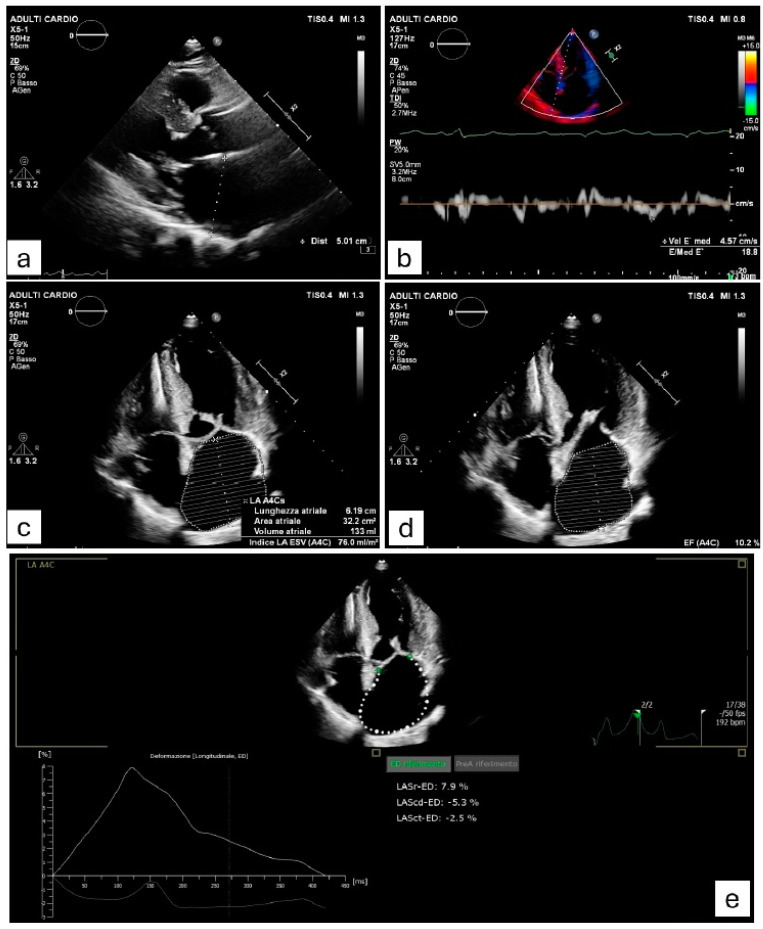
Clinical case of a patient affected by hypertrophic cardiomyopathy (HCM) who experienced a paroxysmal episode of atrial fibrillation (AF). The image shows echocardiographic measurements of various variables which have been demonstrated to be associated with AF onset in HCM. (**a**) Severely increased left atrial antero-posterior diameter (≥45 mm). (**b**) E/e’ ratio ≥ 17, suggesting elevated left ventricular filling pressures. (**c**) Marked dilation of left atrial area (≥28 cm^2^) and left atrial volume index (≥34 mL/m^2^). (**d**) Impaired left atrial ejection fraction (≤45%). (**e**) Alterations in the three left atrial phasic functions (reservoir, conduit, pump), assessed by strain-derived measures.

The left atrium, in healthy individuals, is a thin-walled chamber. In HCM, this structure undergoes structural changes due to heightened afterload [56]. Indeed, LV hypertrophy leads to elevated LV end-diastolic pressure, which is occasionally accompanied by dynamic LV outflow tract (LVOT) obstruction. Wall stress in the LA has been demonstrated to contribute to cardiomyocyte damage and fibrous replacement [57,58]. Furthermore, HCM patients with AF show a greater percentage of LA myocardial fibrosis compared to those without AF, suggesting the use of this parameter as a prognostic indicator of AF onset [59,60]. The concept of atrial cardiomyopathy (ACM) has emerged in recent years, culminating in the publication of the first consensus document in 2016 by four national arrhythmia associations. This document defined ACM as “the presence of structural, architectural, contractile, or electrophysiological alterations in the atria, capable of producing clinically significant consequences” [61]. According to this expert consensus [61], which has been recently updated [54], ACM can be regarded as the pathophysiological basis for the development of atrial arrhythmias and atrial thrombogenesis, which in turn worsen atrial remodeling in a vicious cycle (“AF begets AF”). The atrial mechanical function is divided into three phases during the cardiac cycle [62]: the reservoir, conduit, and contractile phases. Thus, several studies have been conducted with the objective of identifying predictors of ACM, and, consequently of AF, through the use of both echocardiographic and magnetic resonance imaging techniques [63]. In HCM patients, a value of LA antero-posterior diameter ≥ 45 mm is prognostic for higher risk of AF [8,64]. LA diameter measurement is a pragmatic and widely employed approach for evaluating atrial dimensions; however, it is a two-dimensional measurement. Conversely, LA volume index (LAVI) is a more reliable method [65,66]. In a study including 141 HCM patients, a LAVI value ≥ 34 mL/m^2^ was the most sensitive and specific parameter in predicting the development of paroxysmal AF attacks [66]. Maron et al. conducted a prospective evaluation of the prognostic implications of LA structural and functional parameters, as determined through cardiovascular magnetic resonance (CMR) imaging, in a cohort of 427 HCM patients. The authors pioneered the study of left atrial ejection fraction (LAEF), defined as left atrial stroke volume divided by left atrial end-diastolic volume. Their findings revealed that a LAEF < 38%, an increased left atrial end-diastolic volume (≥118 mL), and age ≥ 40 years were independently associated with the development of AF in this population [25]. Losi et al. employed M-mode echocardiography to assess LA function through its global fractional shortening, defined as [LA diameter_max_ − LA diameterdeter_min_)/LA diameter_max_] × 100. The results evidenced that a LA fractional shortening ≤ 16% was an independent predictor of AF. In contrast, the measurements of LA diameter and volume were significantly prognostic for the arrhythmia just when correlated with age [67]. Tuluce et al. demonstrated that, among LA phasic functions, two echocardiographic variables, LATEF (LA total emptying fraction) and LAAEF (LA active emptying fraction), were predictive of AF [18]. Left atrial reservoir function, evaluated through strain-derived measures, is predominantly influenced by atrial compliance [68]. This parameter exhibits a stronger correlation with the stage of atrial remodeling compared to LA size, underscoring the significance of strain parameters in predicting the development of AF. The aforementioned study demonstrated that not only LA reservoir function, but also LA booster pump impairment correlates with the development of AF in HCM patients [69]. The authors also found a significant correlation between higher plasma NT-proBNP levels and worse LA reservoir and booster pump functions [56]. The elevation of serum NT-proBNP—with a cut-off value of >720 pg/mL—was effective in predicting with good accuracy the onset of AF [18]. In another cohort study of 126 patients affected by HCM, Mandeş et al. showed that left atrial booster pump function was the sole LA functional parameter that independently predicted the onset of AF, with a cut-off value of at least −0.88 s^−1^ [14]. In addition to the functional features, the investigation of Doppler parameters in this setting has also been a subject of interest. In a study including 321 patients diagnosed with HCM, Costabel et al. demonstrated an occurrence of AF during follow-up in 11.8% of subjects [10]. This group of patients exhibited an increased size of the LA (area exceeding 28 cm^2^) and a higher E/E’ ratio (>17). In fact, an elevated E/E’ ratio is indicative of elevated ventricular filling pressures, atrial cardiomyopathy, and thus higher predisposition to AF [10]. Figure 2 summarizes the main echocardiographic data found to be significant predictors of AF onset in HCM.

The extension of fibrotic tissue in the left atrium is another typical marker of atrial remodeling. This kind of assessment can be facilitated by late gadolinium enhancement (LGE) on CMR, a method that is both operator-independent and reliable [70,71,72,73,74,75]. In a study by Cochet et al., the pattern of CMR-derived LGE observed at the level of LV-RV junctions was the most accurate parameter associated with the subsequent development of AF [28]. Concurrently, Hohneck et al. observed that—in addition to AF—an extent of LV LGE > 14.4% functioned as an independent predictor of thromboembolic complications in patients with HCM [76]. In agreement, Hollowell et al. retrospectively evaluated 351 HCM patients who underwent CMR. They demonstrated that an extension of left ventricular LGE > 15% was associated with a significant increase in AF occurrence, even in patients with a normal-sized LA (LAVI below 34 mL/m^2^) [77]. Raman et al. evaluated LA function through CMR-derived strain parameters, finding that deformation during the reservoir and booster phases were independent predictors of increased AF risk, regardless of LA size. The authors demonstrated through multivariate analysis that values of LA booster strain ≤ 8% and LA reservoir strain ≤ 18% were each independent determinants of AF. Also, age ≥ 55 years and LAEF ≤ 45% were shown to be significant predictors of AF, in agreement with previous literature [78]. Another parameter that has been investigated is the left atrioventricular coupling index (LACI), defined as the ratio of the indexed volume of the left atrium to the indexed volume of the left ventricle. In a study involving 141 HCM patients in sinus rhythm, Parisi and colleagues studied various imaging parameters regarding the LA, first by echocardiography, then validating them through CMR. The authors found that, among the whole cohort, the 35 patients who developed new-onset AF had significantly greater values of LACI, LA end-diastolic volume, and LA diameter. Specifically, a higher LACI (>44%) and a lower LAEF (<43%) exhibited significant accuracy in predicting the development of AF, even in the absence of substantial LA dilatation [79]. Epicardial adipose tissue (EAT), defined as a visceral fat deposit located near the myocardium and coronary arteries, can be quantified through CMR. This tissue is not only a source of energy, but also associated with pro-inflammatory processes and elevated risk of coronary artery disease. Epicardial fat can infiltrate the atrial wall, promoting atrial structural remodeling and fibrosis. Consequently, the potential role of EAT in the development of AF has been investigated in the general population [80]. More recently, Zhou et al. examined the correlation between the volume of the EAT, assessed by three-dimensional CMR, and the development of AF in 93 patients affected by obstructive HCM. The researchers observed that a higher indexed volume of the EAT (EATVI ≥ 143.8 mL/m^2^) correlated with the development of AF, dyslipidaemia, and also with higher LAVI, left ventricular end-systole volume index, and lower LVEF values. EATVI was found to be an independent predictor of AF at both univariable and multivariable logistic regression models, as well as LAVI and LVEF. The integration of these three parameters yielded superior performance in predicting AF within the study population [81]. Consistent with these findings, Li et al. investigated a larger cohort of HCM patients, demonstrating that EAT volume and EATVI were significantly higher in individuals with AF compared to those without AF. In multivariable analysis, higher LV wall thickness, LA diameter, LAVI, and EATVI were identified as independent predictors of new-onset AF. In this study, EATVI demonstrated better performance compared to the other CMR-derived parameters [82]. Recent advancements in machine learning models for AF risk prediction have shown promising potential in improving early detection and personalized management. These models leverage large datasets from electronic health records, wearable devices, and genetic information to identify high-risk individuals. Key developments include the use of deep learning algorithms to predict AF onset based on ECG data, as well as predictive models that incorporate demographic, clinical, and lifestyle factors. Deep neural networks and convolutional neural networks have been applied to single-lead ECG data for early AF detection. These models excel in handling complex patterns in heart rhythms and can identify subtle markers of AF risk that might be missed by traditional approaches [83]. Advances in wearable devices, like smartwatches, allow continuous monitoring of heart rhythms. Machine learning models can analyze this real-time data to detect arrhythmias and predict AF events before they occur, with high performance [84,85,86,87,88]. Lu and colleagues enrolled a cohort of 1069 patients with HCM and created a machine learning model to predict AF using the following variables: LA volume and diameter, stress and rest LVOT gradient, CMR-derived LGE, peak heart rate during exercise, age at diagnosis, positive genotype, diabetes mellitus, and end-stage renal disease. The area under the receiver-operating curve of this model demonstrated better performance compared to the HCM-AF score [89]. These studies underscore the growing role of machine learning in enhancing AF risk prediction, offering the potential for early intervention and improved patient outcomes.

As discussed in the present paper, Table 2 offers a concise summary of the most significant predictors of AF onset in HCM patients.

In medical practice, an accurate clinical evaluation is pivotal in the assessment of AF risk. In contrast to echocardiographic markers, ECG parameters such as Pdur and PWD are not part of the routine assessment. The most frequently employed are LA measurements; in particular, LAVI represents the most reliable assessment of LA dimension because it is related to body surface area. While the evaluation is typically limited to LA dimensions, the assessment of atrial strain is strongly recommended, particularly in patients who have previously experienced a paroxysmal AF episode [90]. In fact, anatomical remodeling is usually preceded by atrial functional changes. An impairment of these markers can also predict, in addition to AF occurrence, the success of an AF ablation procedure, leading to more accurate selection of patients suitable for this intervention [91]. The recognition of AF predictors enables the development of personalized treatment plans, such as monitoring strategies, anticoagulation therapy, and lifestyle modifications, with the aim of preventing AF-related complications, including stroke. The timely recognition of these predictors is therefore aimed at LA reverse remodeling through timely intervention. Furthermore, the integration of wearable devices and continuous monitoring technologies has the potential to enhance the early detection and proactive management of AF, thereby improving patient outcomes and reducing the burden on healthcare systems [84]. AF is influenced by a wide range of determinants in the general population, including age, hypertension, diabetes, obesity and a history of heart failure. These factors contribute to structural and electrical remodelling of the atria, thereby promoting the onset of AF [92]. AF predictors in the general population are similar to those for the HCM population, and include Pdur and PWD, higher atrial dimensions, impairment of atrial strain functions, and CMR-derived LGE [93]. On the other hand, in HCM there are additional features that predispose the atria to fibrotic remodeling. The structural and functional alterations caused by the hypertrophied myocardium lead to increased atrial pressure, atrial enlargement, and fibrosis, all of which predispose patients to AF. HCM patients are at higher risk for AF compared to the general population. This is further influenced by disease severity and the presence of left ventricular outflow tract obstruction, diastolic dysfunction, and mitral regurgitation [1]. As previously discussed, genetic mutations can also contribute to a higher risk of arrhythmias.

## 6. Limitations

It is important to acknowledge that this review is not systematic, and as such, it possesses certain limitations. The absence of a comprehensive search strategy may result in selection bias, and publication bias could occur by favoring studies with positive results. Additionally, the presence of reviewer bias has the potential to influence the selection of studies included in the review, as well as the interpretation of their findings. Additionally, there are also important limitations in existing studies about the topic discussed in the present review. One major limitation is the relatively small sample sizes in many studies, which can lead to reduced statistical power and may fail to capture the full spectrum of risk factors across diverse populations. Moreover, the findings of these studies are often constrained by the specific characteristics of the study population, such as age, gender, or underlying conditions, making it difficult to generalize the results to broader or more heterogeneous populations. Another key challenge is the limited duration of many studies, which often fail to account for the long-term dynamics of AF risk and progression. Many studies rely on retrospective data, which may introduce biases and fail to capture evolving factors over time. Furthermore, the integration of multiple risk factors (such as lifestyle, genetics, and comorbidities) remains a challenge, as their interactions can be complex and are not always well-represented in the existing literature. To address these challenges, there is a pressing need for larger, multi-center, and prospective trials that can better capture the diversity of populations and provide more robust evidence on the long-term impact of various risk factors. Such studies would also enable more accurate AF prediction and the development of personalized treatment strategies based on real-world data.

## 7. Conclusions

This review analyzes the various risk predictors of AF occurrence in HCM patients, derived by clinical, instrumental, and genetic findings. At this time, there is no evidence to suggest that anticoagulant therapy should be initiated in HCM patients without AF. However, approximately 50% of individuals with HCM experience asymptomatic episodes of AF, which are typically brief and have been linked to adverse outcomes. Several studies aimed to identify risk predictors for new-onset AF in the HCM population, using various techniques. The stratification of AF risk through multiple variables is an invaluable tool for the clinical management of these patients, leading to more closely monitored cases and, consequently, more prompt detection of the arrhythmia. This, in turn, can have a significant impact on the patient’s quality of life and prognosis. Conversely, the identification of patients with a low risk of AF occurrence can provide reassurance to both the patient and the clinician. Further studies are required to assess the potential benefit from anticoagulant prophylaxis in HCM individuals who demonstrate a higher risk of developing AF, even in the absence of documented arrhythmias.

## Figures and Tables

**Table 1:** The most relevant correlations between specific gene mutations and polymorphisms with atrial fibrillation onset in patients affected by hypertrophic cardiomyopathy.

Gene(s) Studied	Protein Encoded	Authors	Main Findings
MYH7 (Arg663His)	β- myosin heavy chain	Gruver et al. [36]	Significantly higher rate of AF onset compared to that of ungenotyped familial HCM population.
MYBPC3, MYH7, other genotypes	myosin binding protein C3, β- myosin heavy chain	Bongini et al. [42]	Three subgroups (MYBPC3, MYH7, and “other genotypes”), with similar LA size: genotype was not correlated to AF onset or severity.
CYP11B2 (344 T > C)	cytochrome P450 11B2	Orenes-Piñero et al. [47]	Independently predicted the onset of AF and was associated with higher aldosterone serum levels.
COL1A1 (2046 G > T)	collagen type I alpha 1 chain	Orenes-Piñero et al. [47]	Not statistically significant trend towards a lower risk of AF onset (potential protective role).
AGTR1 (1166 A > G)	angiotensin II type 1 receptor	Belenkov et al. [48]	Associated with development of HCM with atrial fibrillation.

AF = atrial fibrillation; HCM = hypertrophic cardiomyopathy; LA = left atrium.

**Table 2:** The most significant studies about predictors of atrial fibrillation in patients affected by hypertrophic cardiomyopathy, with their respective cut-off values.

Study	Year	Sample Size	Type of Study	Technique	Significant Variable(s)	Cut-Off Value
Cecchi et al. [15]	1997	110	Retrospective and prospective	ECG	Filtered Pdur	>140 ms
Ozdemir et al. [16]	2004	80	Retrospective	ECG-Echo	P-wave dispersion Filtered Pdur LA diameter	>52.5 ms >134.5 ms >42 mm
Girasis et al. [17]	2013	62	Retrospective, case–control	ECG	LAD-AP LAT peak SR-S Pdur	>42 mm >2.32 s^−1^ >98.8 ms
Mandeş et al. [14]	2022	126	Prospective	ECG-Echo	P-wave dispersion LA booster pump strain	≥47.5 ms ≥−0.88 s^−1^
Tjahiadi et al. [22]	2021	208	Retrospective	ECG-Echo	PA-TDI LA diameter LAVI LA reservoir strain	>115 ms ≥45 mm ≥34 mL/m^2^ <21.3%
Maron et al. [25]	2014	427	Prospective	CMR	Age LAEF LAEDV	>40 years <38% ≥118 ml
Klopotowski et al. [26]	2018	546	Retrospective	Echo	Age Presyncope/syncope/nsVT LA diameter (initially) LA diameter at last follow-up LVEF	>44.5 years Yes >41.5 mm >45.5 mm <65.5%
Olivotto et al. [8]	2001	480	Prospective	Echo	LA diameter	> 45 mm
Tani et al. [66]	2004	141	Retrospective	Echo	LAVI	≥34 mL/m^2^
Losi et al. [67]	2004	150	Prospective	Echo	LA fractional shortening LA diameter LAVI	≤16% ≥45 mm >27 mL/m^2^
Tuluce et al. [56]	2014	70	Prospective	ECG-Echo-biomarkers	P-wave dispersion LATEF LAAEF NT-proBNP	>47.5 ms <49% <36% >720 pg/ml
Costabel et al. [10]	2018	321	Retrospective	Echo	E/e’ LA area	≥17 ≥28 cm^2^
Cochet et al. [28]	2018	209	Prospective	CMR	Fibrosis on RV-LV insertions NYHA class LA volume	
Hohneck et al. [76]	2020	115	Prospective	CMR	LV LGE	>14.4%
Hollowell et al. [77]	2024	351	Retrospective	CMR	LV LGE	>15%
Raman et al. [78]	2021	258	Retrospective	CMR	LA booster strain LA reservoir strain Age LAEF	≤8% ≤18% ≥55 years ≤45%
Parisi et al. [79]	2024	141	Prospective	Echo-CMR	LACI LAEF	>44% <43%
Zhou et al. [81]	2021	93	Retrospective	CMR	EATVI LAVI LVEF	≥143.8 mL/m^2^ ≥69.6 mL/m^2^ ≤64.2%
Li et al. [82]	2024	304	Retrospective	CMR	EATVI LV wall thickness LA diameter LAVI	≥53.9 mL/m^2^ ≥21.7 mm ≥44 mm ≥53.0 mL/m^2^

ECG = electrocardiogram; CMR = cardiac magnetic resonance; Pdur = P-wave duration; LA = left atrium; LAD-AP = Left atrium antero-posterior diameter; LAT peak SR-S = Lateral LA wall peak strain rate in the reservoir period; PA-TDI = peak A wave on tissue Doppler imaging of the atria; LAVI = left atrial volume index; LAEF = left atrial ejection fraction; LAEDV = left atrial end-diastolic volume; nsVT = non-sustained ventricular tachycardia; LVEF = left ventricular ejection fraction; LATEF = LA total emptying fraction; LAAEF = LA active emptying fraction; RV = right ventricle; LV = left ventricle; NYHA = New York Heart Association; LGE = late gadolinium enhancement; LACI = left atrioventricular coupling index; EATVI = epicardial adipose tissue volume index.

## Abbreviations

The following abbreviations are used in this manuscript:

HCM	Hypertrophic cardiomyopathy
LV	Left ventricle
AF	Atrial fibrillation
ECG	Electrocardiogram
Pdur	P-wave duration
PWD	P-wave dispersion
Pdurmax	Maximum P-wave duration
LA	Left atrium
BMI	Body mass index
NYHA	New York Heart Association
HF	Heart failure
CV	Cerebrovascular accidents
β-MHC	β-cardiac myosin heavy chain
RAAS	Renin–angiotensin–aldosterone system
LVOT	Left ventricular outflow tract
ACM	Atrial cardiomyopathy
LAVI	Left atrial volume index
CMR	Cardiovascular magnetic resonance
LAEF	Left atrial ejection fraction
LATEF	Left atrial total emptying fraction
LAAEF	Left atrial active emptying fraction
LGE	Late gadolinium enhancement
LACI	Left atrioventricular coupling index
EAT	Epicardial adipose tissue
